# Measurement of the distance between corneal apex and pupil center in patients following small-incision lenticule extraction or implantable collamer lens implantation and its correlation with the surgical-induced astigmatism

**DOI:** 10.1186/s12886-024-03352-6

**Published:** 2024-03-07

**Authors:** Yishan Qian, Lan Ding, Yanlan Ding, Lin Jiang, Zesheng Liu, Xingtao Zhou

**Affiliations:** 1 Department of Ophthalmology, Eye and ENT Hospital, Fudan University, Shanghai, People’s Republic of China; 2 NHC Key Laboratory of Myopia, Fudan University, 83th Fenyang Rd, Shanghai, 200031 People’s Republic of China

**Keywords:** Angle Kappa, Small-incision lenticule extraction, Implantable collamer lens

## Abstract

**Background:**

To investigate the change in the distance between corneal apex and pupil center after small-incision lenticule extraction (SMILE) or implantable collamer lens (ICL) implantation and its correlation with surgical-induced astigmatism (SIA).

**Methods:**

This study included patients who had undergone SMILE (*n* = 112) or ICL implantation (*n* = 110) to correct myopia and myopic astigmatism. The angle kappa was measured using a Scheimpflug imaging device (Pentacam) and represented as Cartesian values between the pupil center and the corneal vertex (X, Y) and chord u ($\sqrt{{X}^{2}+{Y}^{2}}@$orientation), and was compared pre- and post-operative.

**Results:**

Following SMILE, the magnitude of chord u$(\sqrt{{X}^{2}+{Y}^{2}}$) significantly increased in both eyes (Wilcoxon signed-rank test, OD: *P*<0.001; OS: *P*=0.007), while no significant change was observed in the orientation. A significant correlation was found between the J_0_ component of SIA and the change in the magnitude of chord u for both eyes (OD: R^2^=0.128, *P*<0.001; OS: R^2^=0.033, *P*=0.004). After ICL implantation, the orientation of the chord u was significantly different in the right eye (Wilcoxon signed-rank test, *P* = 0.008), and the Y-intercept significantly decreased in both eyes (Wilcoxon signed-rank test, *P*<0.001). A significant correlation was found between J_0_ of SIA and the change in the magnitude of chord u for the right eyes (R^2^=0.066, *P*=0.002). A significant correlation was found between J_45_ of SIA and the change in the magnitude of chord u for the left eyes (R^2^=0.037, *P*=0.044).

**Conclusions:**

The magnitude of the chord u increased following the SMILE procedure, whereas the Y-intercept significantly decreased after ICL implantation. SIA was related to the change in the magnitude of chord u.

## Background

Angle kappa represents the angle between the visual axis (the line that connects the fixation point of the fovea and passes through the nodal point of the eye) and pupillary axis (the line passing through the center of the pupil and perpendicular to the cornea) due to the displacement of the fovea to the pupillary axis [1]. As the visual axis is a theoretical assumption, clinically, the angle kappa is estimated as the angular distance between the line of sight (the line that connects the fixation point and the entrance pupil) and the pupillary axis or as the X and Y Cartesian values of the chord u which represents the decentration between the pupil center and the corneal vertex [2, 3]. An accurate measurement of angle kappa is crucial in the preoperative assessment of kerato-refractive and cataract surgery. Previous studies have confirmed that the repeatability and reproducibility of the measurements for angle kappa are high for the same device, whereas different devices could produce slightly different results owing to different principles [4–7].

Although many studies exist on the measurement of angle kappa before refractive surgeries and on the influence of angle kappa on the visual outcomes of corneal refractive surgery, only a few studies have measured angle kappa following refractive surgeries [8, 9]. A postoperative change in angle kappa may impact the enhancement procedures or multifocal intraocular lens (MIOL) implantations in eyes with a history of refractive surgery. This study employed a Pentacam tomography system (Oculus GmbH, Wetzlar, Germany) to investigate the changes in the decentration between the pupil center and the corneal vertex after two types of most often performed refractive surgery: small-incision lenticule extraction (SMILE) and implantable collamer lens (ICL) implantation.

## Methods

This retrospective study included patients who underwent SMILE or ICL implantation for myopia and myopic astigmatism correction between December 2021 and May 2022. Inclusion criteria were the following: patients between 18 and 45 years; the sum of sphere and astigmatism less than − 10.0D for SMILE or -18.0D for ICL; best-corrected distance visual acuity (BCVA) better than 20/25; stable refraction for 2 years before surgery; and the absence of other pathologic ocular conditions or relevant systemic diseases. Only patients with an anterior chamber depth of 2.8 mm or greater and a preoperative endothelial cell density of 2000 cells/mm^2^ or greater were eligible for ICL implantation. This study followed the tenets of the Declaration of Helsinki and was approved by the Ethics Committee of the EENT Hospital of Fudan University. Written informed consent was obtained from all the patients.

All patients underwent a comprehensive ophthalmological examination, including uncorrected and BCVA, objective and subjective refraction, tomography, corneal endothelial microscopy, pupil diameter (PD), intraocular pressure, slit-lamp anterior segment evaluation, and fundus examination with dilated pupils.

### Measurements of angle kappa and vector analysis for astigmatism

The angle kappa was measured using a Pentacam (Oculus GmbH, Wetzlar, Germany) by the same experienced examiner under the same darkened ambient light conditions. Patients were asked to look at the fixation target, and an automatic release mode was used. Angle kappa was represented in Cartesian coordinates (X, Y), where X indicates the x coordinate of the pupil center relative to the corneal vertex, and Y indicates the y coordinate of the pupil center relative to the corneal vertex. According to Chang et al., the displacement between the corneal vertex and pupil center is termed chord u [3]. The chord length was calculated as $\sqrt{{X}^{2}+{Y}^{2}}$, and the orientation of chord u was calculated on the basis of the X and Y with the range of 0–360°. The postoperative change in the magnitude of chord u was calculated as $\sqrt{{({X}_{1}-{X}_{2})}^{2}+{({Y}_{1}-{Y}_{2})}^{2}}$. Our previous study proved the reproducibility of angle kappa measurements made by the Pentacam with an intraclass correlation coefficient (ICC) value of 0.911 [10]. Two consecutive measurements in each eye were performed preoperatively and 3 months postoperatively. Make sure that the difference in pupil diameter is within ± 0.5 mm at each measurement for the same eye. Eyes were grouped according to their preoperative coordinates (X and Y). Those with X and Y-intercepts smaller than 0.2 mm were defined as small angle kappa group (SAK group), and those with either X or Y-intercepts larger than 0.2 mm were defined as large angle kappa group (LAK group).

Vector analysis for astigmatism was performed using the method developed by Alpins et al. [11] Surgical-induced astigmatism (SIA) is the amount and axis of astigmatic change caused by surgery. It was determined as the vector difference between the pre- and postoperative astigmatism determined by manifest refraction. Two cross-cylinder components, J_0_ and J_45_, are defined as follows: *J*_*0*_*=-C/2cos(2α)* and *J*_*45*_*=-C/2sin(2α)*, where C is the magnitude of cylinder power present and α is the axis in radians. J_0_ represents the horizontal/vertical component of astigmatism, and J_45_ represents astigmatism with axes at 45° and 135°.

### Surgical technique

All SMILE procedures were performed by an experienced surgeon (YS, Qian) using a VisuMax femtosecond laser system (Carl Zeiss Meditec AG, Jena, Germany) with a repetition rate of 500 kHz and pulse energy of 130 nJ. We used the centration technique described by Liu et al. [12] Briefly, a centration map was drawn for each patient according to the angle kappa data from Pentacam. During surgery, the patient was asked to focus on the fixation light before the initiation of docking and suction. Centration is accepted when the desired corneal vertex location (shown in the centration map) superimposes the center of the “touch zone.” Following the incision, the refractive lenticule was dissected, separated through a 2 mm-side incision, and manually removed. All ICL surgeries were performed by an experienced surgeon using the same technique (XT, Zhou). Standard ICL surgery was performed, and a 3-mm corneal incision was made using the procedure described in our previous report. For the toric implantable collamer lens (TICL), preoperative corneal marking of the desired axis was performed by the surgeon using a slit lamp with two opposing marks placed using a marking pen. No intraoperative or postoperative complications were observed in patients who underwent SMILE or ICL implantation.

### Statistical analysis

Statistical analyses were performed using SPSS software (version 23.0; SPSS Inc., Chicago, IL, USA). All data are reported as mean ± standard deviation. Statistical significance was set at *P* < 0.05. Data from the left and right eyes were analyzed separately. The Kolmogorov-Smirnov test was used to confirm the normality of the data. The paired t-test was used for normally distributed parameters to assess the difference between pre and postoperative data, and the Wilcoxon signed-rank test was used for non-normally distributed parameters. The relationship between SIA and changes in the magnitude of chord u was explored using Spearman’s correlation.

## Results

**Fig. 1:**
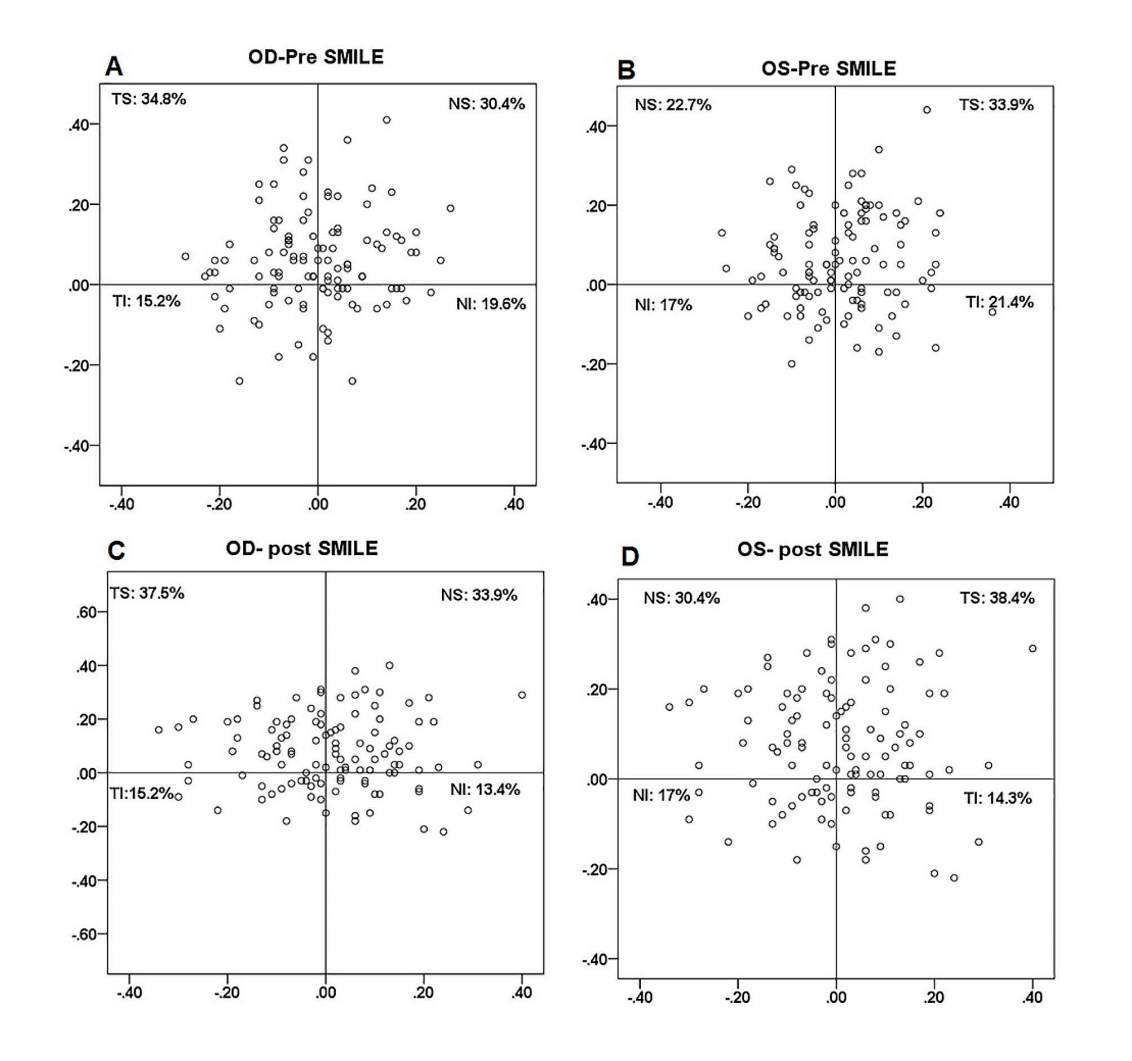
Distribution of the pupil centers relative to the corneal vertex before and after small-incision lenticule extraction (SMILE) for the right eye (OD: **A**, **C**) and left eye (OS: **B**, **D**)

**Fig. 2:**
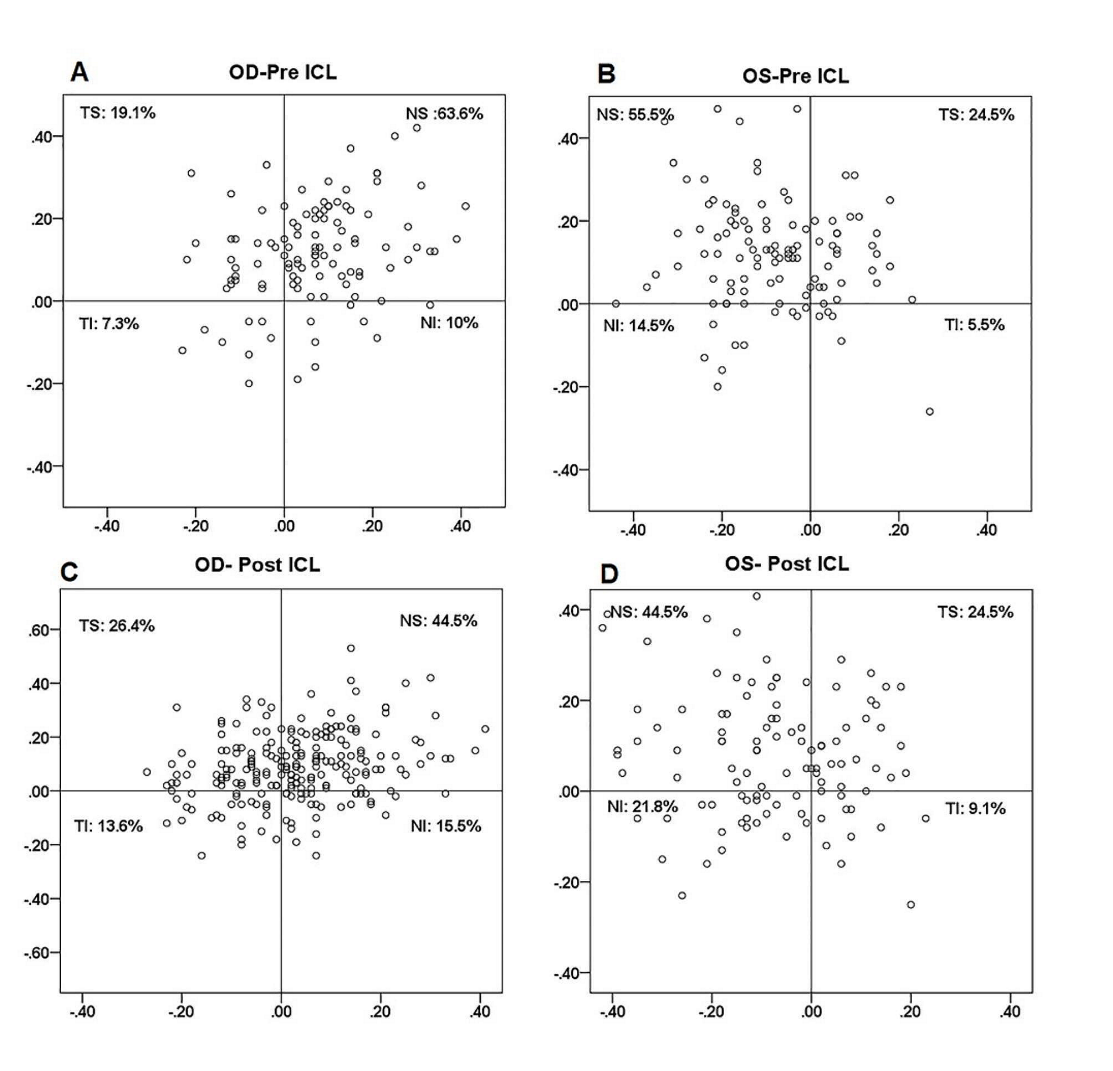
Distribution of the pupil centers relative to the corneal vertex before and after implantable collamer lens (ICL) implantation for the right eye (OD: **A**, **C**) and left eye (OS: **B**, **D**)

The study included 444 eyes of 222 patients (SMILE, *n* = 112; ICL, *n* = 110). Data from the left and right eyes were analyzed and reported separately. The mean age was 32.9 ± 9.0 years for patients undergoing the SMILE procedure and 32.7 ± 6.9 years for patients undergoing ICL implantation. The basic characteristics of the patients are summarized in Table 1. No significant difference was observed in PD after SMILE (Wilcoxon signed-rank test, *P* > 0.05), whereas PD was greater after ICL implantation (Wilcoxon signed-rank test, *P* < 0.001). The distribution of the pupil center relative to the corneal vertex is illustrated in Fig. 1 (SMILE) and Fig. 2 (ICL). For the SMILE eyes, the mean preoperative magnitude of chord u was 0.153 ± 0.086 mm for the right eye and 0.161 ± 0.103 mm for the left eye. A total of 76.8% (86/112) of the right eyes were classified as small angle kappa group (SAK group), and 23.2% (26/112) as large angle kappa group (LAK group); 80.4% (90/112) of the left eyes were classified as SAK group and 19.6% (22/112) as LAK group. The pupil centers were commonly located in the temporal-superior (TS) quadrant of the corneal vertex. For the ICL patients, the mean preoperative magnitude of chord u was 0.205 ± 0.106 mm for the right eye and 0.208 ± 0.113 mm for the left eye. Of the right eyes, 62.7% (69/110) were classified as SAK group, and 27.3% (31/110) were classified as LAK group; 65.5% (72/110) of the left eyes were classified as SAK group, and 34.5% (28/110) were classified as LAK group. The pupil centers were commonly located in the nasal-superior (NS) quadrant of the corneal vertex.

**Table 1:** Basic characteristics of the patients

	Preoperative			Postoperative		
	SE (D)	PD (mm)	CDVA (LOGMAR)	SE (D)	PD (mm)	UDVA (LOGMAR)
**SMILE (** ***n*** ** = 112)**						
Right	-4.32 ± 1.56 (-1.25, -9.0)	3.27 ± 0.59 (2.2, 5.0)	-0.01 ± 0.04 (-0.08, 0.10)	0.05 ± 0.32 (-1.25, 1)	3.11 ± 0.51 (2.1, 4.6)	-0.004 ± 0.10 (-0.18, 0.2)
Left	-4.25 ± 1.55 (-1.0, -8.5)	3.16 ± 0.54 (2.2, 4.8)	-0.02 ± 0.05 (-0.18, 0)	0.1 ± 0.27 (-0.75, 0.75)	3.23 ± 0.53 (2.1, 4.8)	-0.005 ± 0.07 (-0.18, 0.30)
**ICL (** ***n*** ** = 110)**						
Right	-9.04 ± 2.75 (-17.0, -3.0)	3.09 ± 0.58 (1.9, 4.5)	0.04 ± 0.06 (-0.08, 0.30)	-0.20 ± 0.29 (-1.0, 0.25)	3.21 ± 0.59 (2.0, 4.8)	0.04 ± 0.12 (-0.08, 0.30)
Left	-10.11 ± 3.10 (-17.5, -2.5)	3.02 ± 0.53 (1.8, 4.6)	0.05 ± 0.07 (-0.08, 0.30)	-0.08 ± 0.27 (-1.5, 0.75)	3.10 ± 0.59 (1.9, 4.6)	0.006 ± 0.13 (-0.18, 0.40)

CDVA = corrected distant vision acuity; ICL = implantable collamer lens; PD = pupil diameter; SE = spherical equivalent; SMILE = small-incision lenticule extraction; UDVA = uncorrected distant vision acuity

### Angle kappa measurements after small-incision lenticule extraction

The magnitude of the chord u significantly increased after SMILE procedures in both eyes (Wilcoxon signed-rank test, OD: *P* < 0.001; OS: *P* = 0.007, Table 2). No significant difference was found in the orientation of the chord u after SMILE (Wilcoxon signed-rank test, *P* > 0.05, Fig. 1). The Y-intercept significantly increased in the right eye (Wilcoxon signed-rank test, *P* = 0.006). After SMILE, the pupil center remained in the same quadrant relative to the corneal vertex in 63.4% (71/112) of the right eye and 68.8% (77/112) of the left eye. No significant difference was found in the quadrantal distributions before and after the SMILE procedure (Pearson Chi-square: OD:*P* = 0.646; OS:*P* = 0.563).

**Table 2:** Comparison of chord u measurements before and after small-incision lenticule extraction (SMILE) surgery

	Preoperative	Postoperative	*P* ^#^
**OD (** ***n*** ** = 112)**			
Magnitude (mm)	0.153 ± 0.086 (0.01, 0.43)	0.188 ± 0.097 (0.01, 0.60)	< 0.001
Orientation (°)	152.9 ± 101.2 (13, 357)	141.0 ± 92.7 (0, 354)	0.111
X-intercept (mm)	-0.003 ± 0.114 (-0.27, 0.27)	-0.009 ± 0.135 (-0.31, 0.31)	0.502
Y-intercept (mm)	0.06 ± 0.12 (-0.24, 0.41)	0.08 ± 0.14 (-0.22, 0.59)	0.006
**OS (** ***n*** ** = 112)**			
Magnitude (mm)	0.161 ± 0.103 (0.01, 0.74)	0.186 ± 0.107 (0.02, 0.62)	0.007
Orientation (°)	152.9 ± 101.7 (0, 357)	137.0 ± 98.3 (0, 342)	0.558
X-intercept (mm)	0.008 ± 0.129 (-0.60, 0.36)	0.005 ± 0.145 (-0.50, 0.40)	0.757
Y-intercept (mm)	0.055 ± 0.13 (-0.44, 0.44)	0.069 ± 0.143 (-0.36, 0.40)	0.092

^#^ Wilcoxon signed-rank test

**Fig. 3:**
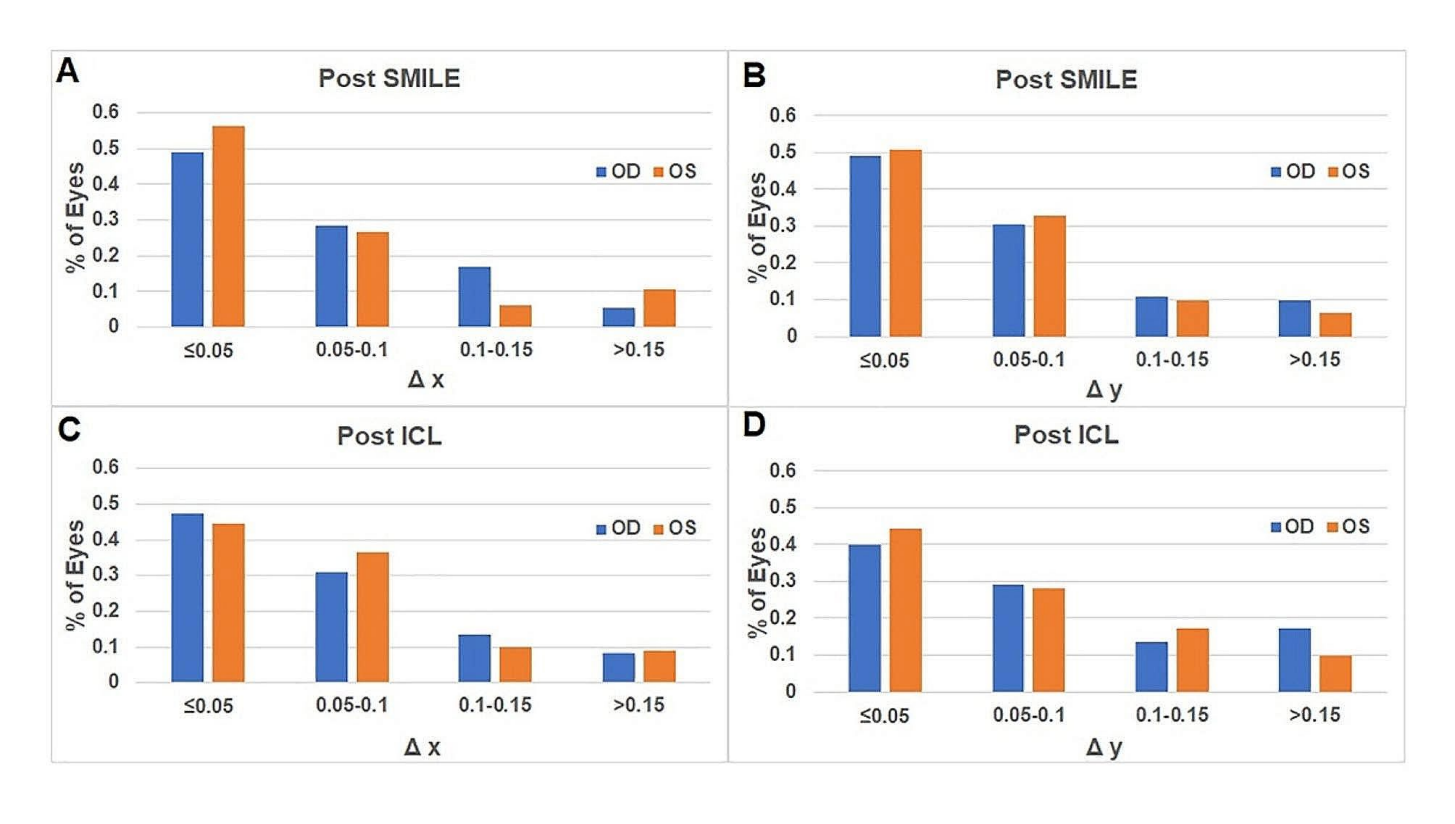
The changes in the X (ΔX) or Y (ΔY) intercepts between pre and postoperative measurements for small-incision lenticule extraction (SMILE group: **A**, **B**) and implantable collamer lens (ICL group: **C**, **D**). OD = right eye; OS = left eye

Figure 3 illustrates the distributions of the change in X-intercepts (ΔX=∣preoperative X-postoperative X∣) and Y-intercepts (ΔY=∣preoperative Y-postoperative Y∣) after SMILE. Approximately 22.4% of the ΔX were greater than 0.1 mm for the right eyes, 17% of the ΔX were greater than 0.1 mm for the left eyes; 20.5% of the ΔY were greater than 0.1 mm for the right eyes, and 16.1% of the ΔY were greater than 0.1 mm for the left eyes.

When the eyes were further grouped into the SAK group and LAK group, the magnitude of the chord u significantly increased after SMILE only in the SAK group for both eyes (Wilcoxon signed-rank test, OD: *P* < 0.001; OS: *P* = 0.001, Table 3) and the Y-intercept significantly increased in the SAK group for the right eye (Wilcoxon signed-rank test, OD: *P* = 0.006).

**Table 3:** Comparison of chord u measurements before and after small-incision lenticule extraction (SMILE) surgery in the small and large angle kappa groups

	Preoperative	Postoperative	*P* ^#^
**OD-small (** ***n*** ** = 86)**			
Magnitude (mm)	0.118 ± 0.059 (0.01, 0.24)	0.163 ± 0.082 (0.01, 0.38)	< 0.001
Orientation (°)	160.5 ± 106.7 (13, 357)	145.6 ± 96.4 (0, 354)	0.155
X-intercept (mm)	0.004 ± 0.097 (-0.20, 0.20)	-0.008 ± 0.130 (-0.31, 0.29)	0.373
Y-intercept (mm)	0.029 ± 0.084 (-0.18, 0.20)	0.052 ± 0.119 (-0.22, 0.37)	0.006
**OD-large (** ***n*** ** = 26)**			
Magnitude (mm)	0.270 ± 0.055 (0.21, 0.43)	0.268 ± 0.101 (0.07, 0.60)	0.809
Orientation (°)	127.8 ± 76.9 (14, 355)	125.7 ± 78.9 (10, 349)	0.657
X-intercept (mm)	-0.027 ± 0.157 (-0.27, 0.27)	-0.012 ± 0.152 (-0.26, 0.31)	0.423
Y-intercept (mm)	0.154 ± 0.169 (-0.24, 0.41)	0.169 ± 0.179 (-0.21, 0.59)	0.553
**OS-small (** ***n*** ** = 90)**			
Magnitude (mm)	0.126 ± 0.061 (0.01, 0.23)	0.160 ± 0.092 (0.02, 0.49)	0.001
Orientation (°)	160.2 ± 100.8 (0, 352)	145.8 ± 102.2 (0, 342)	0.323
X-intercept (mm)	0.001 ± 0.094 (-0.20, 0.20)	0.004 ± 0.131 (-0.34, 0.40)	0.628
Y-intercept (mm)	0.033 ± 0.099 (-0.20, 0.20)	0.044 ± 0.123 (-0.22, 0.31)	0.268
**OS-large (** ***n*** ** = 22)**			
Magnitude (mm)	0.305 ± 0.115 (0.22, 0.74)	0.293 ± 0.101 (0.16, 0.62)	0.527
Orientation (°)	123.2 ± 102.5 (8, 357)	100.9 ± 71.5 (3, 334)	0.322
X-intercept (mm)	0.038 ± 0.223 (-0.60, 0.36)	0.012 ± 0.196 (-0.50, 0.31)	0.119
Y-intercept (mm)	0.144 ± 0.193 (-0.44, 0.44)	0.171 ± 0.174 (-0.36, 0.40)	0.158

^#^ Wilcoxon signed-rank test

### Angle kappa measurements following implantable collamer lens implantation

No significant difference was found in the magnitude of the chord u after ICL implantation in either eye (Table 4; Fig. 2). The orientation of chord u differed for the right eye (Wilcoxon signed-rank test, *P* = 0.008,). The Y-intercept significantly decreased in both eyes after ICL implantation (Wilcoxon signed-rank test, *P* < 0.001). After ICL implantation, the pupil center remained in the same quadrant relative to the corneal vertex in 66.4% (73/110) of the right eye and 70% (77/110) of the left eye. The quadrantal distribution of the angle kappa differed after ICL implantation in the right eye (Pearson Chi-square: OD: *P* = 0.038; OS, *P* = 0.271).

**Table 4:** Comparison of chord u measurements before and after implantable collamer lens (ICL) implantation

	Preoperative	Postoperative	*P* ^#^
**OD (** ***n*** ** = 110)**			
Magnitude (mm)	0.205 ± 0.106 (0.04, 0.55)	0.208 ± 0.127 (0.02, 0.65)	0.972
Orientation (°)	100.5 ± 84.0 (0, 358)	133.1 ± 107.6 (0, 358)	0.008^*^
X-intercept (mm)	0.067 ± 0.136 (-0.23, 0.41)	0.052 ± 0.163 (-0.33, 0.54)	0.068
Y-intercept (mm)	0.118 ± 0.129 (-0.20, 0.53)	0.078 ± 0.155 (-0.29, 0.64)	< 0.001
**OS (** ***n*** ** = 110)**			
Magnitude (mm)	0.208 ± 0.113 (0.01, 0.55)	0.202 ± 0.121 (0.02, 0.59)	0.26
Orientation (°)	129.2 ± 65.2 (0, 333)	144.6 ± 80.5 (0, 351)	0.14
X-intercept (mm)	-0.079 ± 0.138 (-0.44, 0.27)	-0.076 ± 0.153 (-0.42, 0.23)	0.333
Y-intercept (mm)	0.118 ± 0.131 (-0.26, 0.47)	0.081 ± 0.143 (-0.25, 0.52)	< 0.001

^#^ Wilcoxon signed-rank test

Approximately 21.8% of the ΔX was greater than 0.1 mm for the right eyes, 19.1% of the ΔX was greater than 0.1 mm for the left eyes; 30.9% of the ΔY was greater than 0.1 mm for the right eyes, and 27.3% of the ΔY was greater than 0.1 mm for the left eyes (Fig. 3). The orientation of the postoperative chord u differed after ICL implantation in the SAK group for the right eye (Wilcoxon signed-rank test, *P* = 0.015, Table 5). The magnitude of the chord u significantly decreased in the LAK group for the left eye (Wilcoxon signed-rank test, *P* = 0.007). The Y-intercepts significantly decreased in both groups and eyes (Table 5).

**Table 5:** Comparison of chord u measurements before and after implantable collamer lens (ICL) implantation in the small and large angle kappa groups

	Preoperative	Postoperative	*P* ^#^
**OD-small (** ***n*** ** = 69)**			
Magnitude (mm)	0.140 ± 0.049 (0.04, 0.24)	0.146 ± 0.079 (0.02, 0.33)	0.754
Orientation (°)	114.0 ± 87.7 (4, 356)	151.6 ± 108.0 (0, 356)	0.015^*^
X-intercept (mm)	0.020 ± 0.096 (-0.20, 0.18)	0.005 ± 0.121 (-0.29, 0.30)	0.140
Y-intercept (mm)	0.063 ± 0.094 (-0.20, 0.20)	0.017 ± 0.115 (-0.29, 0.33)	< 0.001
**OD-large (** ***n*** ** = 41)**			
Magnitude (mm)	0.315 ± 0.085 (0.22, 0.55)	0.310 ± 0.126 (0.09, 0.65)	0.737
Orientation (°)	77.9 ± 72.9 (0, 358)	102.0 ± 100.7 (7, 358)	0.288
X-intercept (mm)	0.145 ± 0.159 (-0.23, 0.41)	0.132 ± 0.193 (-0.33, 0.54)	0.299
Y-intercept (mm)	0.212 ± 0.127 (-0.12, 0.53)	0.179 ± 0.163 (-0.18, 0.64)	0.016
**OS-small (** ***n*** ** = 72)**			
Magnitude (mm)	0.145 ± 0.064 (0.01, 0.27)	0.156 ± 0.087 (0.02, 0.40)	0.503
Orientation (°)	127.0 ± 70.7 (0, 333)	142.7 ± 88.2 (0, 351)	0.270
X-intercept (mm)	-0.041 ± 0.099 (-0.20, 0.18)	0.040 ± 0.126 (-0.39, 0.19)	0.466
Y-intercept (mm)	0.083 ± 0.083 (-0.16, 0.20)	0.049 ± 0.112 (-0.23, 0.29)	< 0.001
**OS-large (** ***n*** ** = 38)**			
Magnitude (mm)	0.328 ± 0.085 (0.22, 0.55)	0.291 ± 0.128 (0.05, 0.59)	0.007
Orientation (°)	133.4 ± 53.8 (2, 316)	148.2 ± 64.2 (29, 345)	0.286
X-intercept (mm)	-0.151 ± 0.171 (-0.44, 0.27)	-0.143 ± 0.176 (-0.42, 0.23)	0.519
Y-intercept `	0.185 ± 0.174 (-0.26, 0.47)	0.142 ± 0.174 (-0.25, 0.52)	0.002

^#^ Wilcoxon signed-rank test

### Association between surgically-induced astigmatism and changes in the magnitude of chord u

**Fig. 4:**
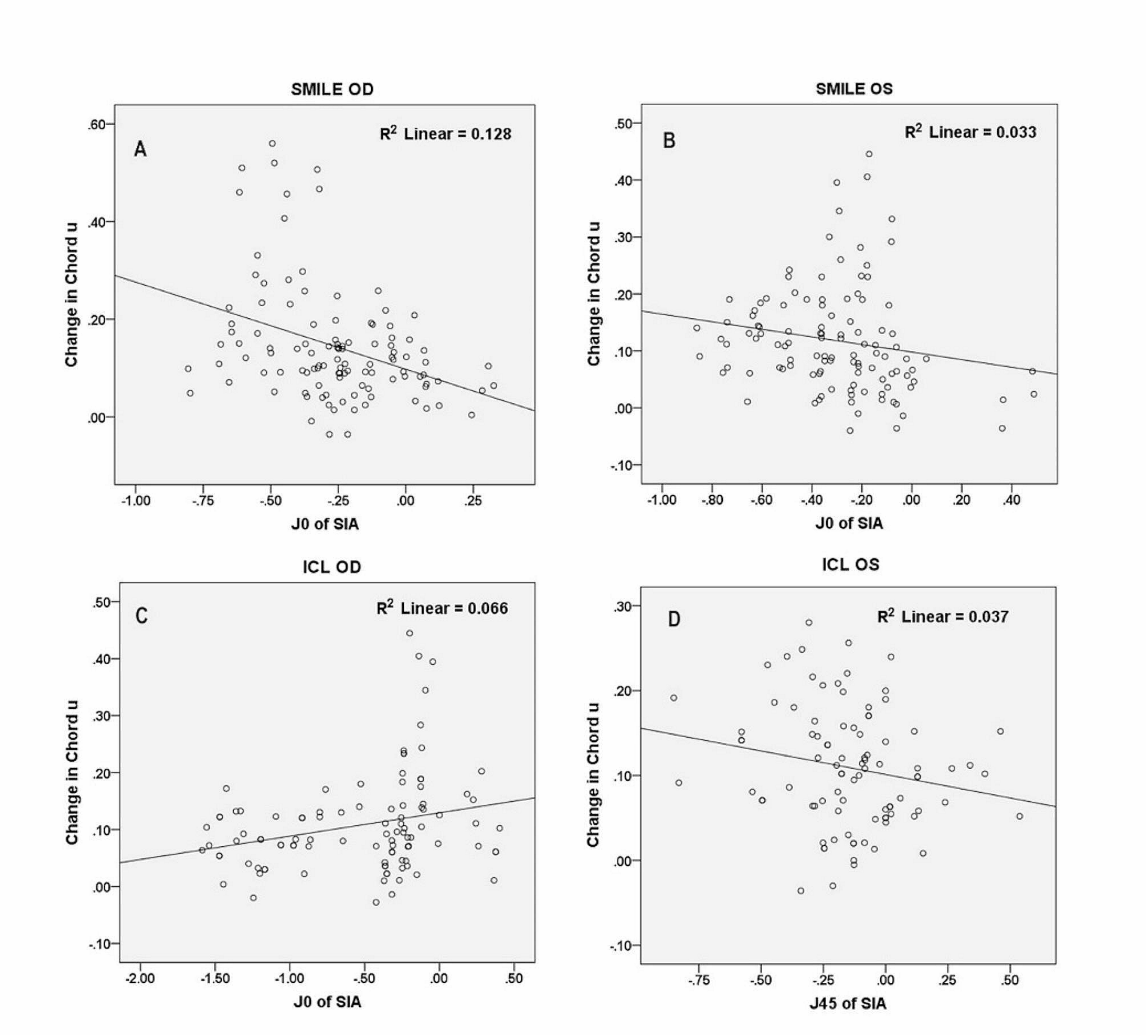
Distribution of the surgically-induced astigmatism (J_0_ or J_45_) and change in the magnitude of chord u after small-incision lenticule extraction (SMILE: **A**, **B**) or implantable collamer lens implantation (ICL: **C**, **D**). Only those having statistical significance in the correlation analyses are displayed here. OD = right eye; OS = left eye

After SMILE procedures, a significant correlation was found between the J_0_ of SIA and the postoperative change in the magnitude of chord u for both eyes (OD: R^2^ = 0.128, *P* < 0.001; OS: R^2^ = 0.033, *P* = 0.004, Fig. 4A and B). No significant correlation was found between the J_45_ of SIA and the postoperative change in the magnitude of chord u (OD: *P* = 0.321; OS: *P* = 0.192). Following ICL implantation, significant correlation was found between the J_0_ of SIA and the postoperative change in the magnitude of chord u for the right eyes (OD: R^2^ = 0.066, *P* = 0.002, Fig. 4C; OS: R^2^ = 0.042, *P* = 0.203). A significant correlation was found between the J_45_ of SIA and the postoperative change in the magnitude of chord u for the left eyes (OD: R^2^ = 0.018, *P* = 0.289; OS: R^2^ = 0.037, *P* = 0.044, Fig. 4D).

## Discussion

This study found that the magnitude of the chord u significantly increased in both eyes following SMILE and significantly correlated with the J_0_ component of SIA. After ICL implantation, the orientation of the angle kappa was significantly different in the right eye. The Y-intercept decreased significantly in both eyes after ICL implantation. A significant correlation was found between the J_0_ component of SIA and the change in the magnitude of the chord u for the right eye. A significant correlation was found between the J_45_ component of SIA and the change in the magnitude of the chord u for the left eye.

A review of the literature revealed that few studies exist on postoperative changes in the angle kappa. Zarei-Ghanavati et al. evaluated the changes in the mean angle kappa after photorefractive keratectomy (PRK) for the correction of myopia. They found no difference in the magnitude of angle kappa or its corneal intercepts post-PRK [8]. Ding et al. investigated the angle kappa changes after sub-Bowman-keratomileusis (SBK) and found that the angle kappa decreased in patients with a large preoperative angle kappa and increased in patients with a small preoperative angle kappa [9]. Wang et al. employed iTrace to observe the distributions and changes of the angle kappa in patients undergoing cataract surgery. They found that the magnitude of angle kappa significantly decreased after phacoemulsification, whereas no significant difference was observed in the orientation of angle kappa [13]. In this study, we found that the magnitude of angle kappa significantly increased in patients with small angle kappa and decreased in patients with large angle kappa after SMILE. The visual axis has been reported to change in patients after surgeries for cortical or localized subcapsular cataracts; however, no evidence supports that the visual axis could change following refractive surgeries. One possible reason for the change in angle kappa following refractive surgery could be a shift in the center of the pupil. The virtual image of the pupil (the entrance pupil) depends upon the anatomical and optical properties of the eye [14]. The pupil outline could be distorted or dislocated due to optical aberration of the cornea [15]. Moreover, the level of aberration in an eye has been reported to affect pupil size. The pupil size was adjusted to provide good image quality on the retina by controlling image sharpness and illuminance [16].

The effect of changes in angle kappa on postoperative optical and visual performance remains unclear. The correlation between SIA and change in the magnitude of chord u found in this study suggests that a reduction in refractive astigmatism following refractive surgery could have an impact on the relative locations of the corneal apex and pupil center. Tutchenko et al. found that changes in the location of the first Purkinje image relative to the pupil center after phacoemulsification correlated to changes in refractive astigmatism (the J_45_ vector), especially when the IOL is aspheric [17]. Although no studies exist on the relationship between angle kappa and visual function after ICL implantation, the change in angle kappa apart from ICL tilt or decentration could be one of the causes of photostress after ICL implantation [18, 19]. Further studies are needed to clarify this question. Moreover, the elevation maps obtained by the Pentacam system using the best-fit sphere (BFS) might be affected by large-angle kappa, which could confound the assessment of refractive surgery candidates [20]. Therefore, the change in angle kappa after corneal refractive surgery should be considered when evaluating postoperative ectasia.

The rapid growth of refractive surgery over the last few decades has created new challenges for ophthalmologists. For example, the main challenge of cataract surgery in patients with a history of refractive surgery is the optimization of intraocular lens (IOL) implantation to achieve the desired refractive outcome, especially for MIOL. Previous studies have found that ocular biometric measurements, such as axial length, anterior chamber depth, mean keratometry, white to white, and central corneal thickness, changed significantly after ICL implantation [21–23]. The change in angle kappa found in this study should also be considered a parameter during planning for MIOL. Similarly, the change in angle kappa should be considered in enhancement procedures of refractive surgeries.

This study has limitations. First, the angle kappa was measured by Pentacam and represented as X–Y Cartesian coordinates between the corneal vertex and pupil center. Further study would use other devices such as iTrace, which displays X–Y Cartesian coordinates between the visual axis and pupil center [24]. Second, the pupil center was located more commonly at the temporal side of the vertex in the SMILE group, which was consistent with those obtained by other studies [10, 24]. However, the pupil center was located more commonly at the nasal side of the corneal vertex in the ICL group. This discrepancy may be related to the predominance of high myopia in the ICL group. Further studies investigating the distribution of the angle kappa in patients with high myopia using different devices would help resolve this question. Third, there is no control group who did not undergo refractive surgeries in this study because two consecutive measurements of the angle kappa were performed preoperatively in each eye and demonstrated good reproducibility.

## Conclusions

In summary, we found that the magnitude of the chord u increased following the SMILE procedure, whereas the Y-intercept significantly decreased after ICL implantation. The correlation between surgically induced astigmatism and change in the magnitude of chord u found in this study suggests that a reduction in refractive astigmatism following refractive surgery has an impact on the distance between the corneal apex and pupil center.

## Data Availability

The datasets used and/or analyzed during the current study are available from the corresponding author upon reasonable request.

## Abbreviations

BCVA: Best-corrected distance visual acuity
BFS: Best-fit sphere
ICL: Implantable collamer lens
LAK: Large angle kappa
MIOL: Multifocal intraocular lenses
PD: Pupil diameter
PRK: Photorefractive keratectomy
SAK: Small angle kappa
SBK: Sub-Bowman-keratomileusis
SMILE: Small-incision lenticule extraction
